# Severe Acute Respiratory Syndrome–associated Coronavirus Infection

**DOI:** 10.3201/eid0911.030421

**Published:** 2003-11

**Authors:** Paul K.S. Chan, Margaret Ip, KC Ng, Rickjason C. W. Chan, Alan Wu, Nelson Lee, Timothy H. Rainer, Gavin M. Joynt, Joseph J. Y. Sung, John S. Tam

**Affiliations:** * The Chinese University of Hong Kong, Prince of Wales Hospital, Shatin, Hong Kong

**Keywords:** Coronavirus, health care worker, hospital, prevalence, severe acute respiratory syndrome, SARS

## Abstract

Whether severe acute respiratory syndrome–associated coronavirus (SARS-CoV) infection can be asymptomatic is unclear. We examined the seroprevalence of SARS-CoV among 674 healthcare workers from a hospital in which a SARS outbreak had occurred. A total of 353 (52%) experienced mild self-limiting illnesses, and 321 (48%) were asymptomatic throughout the course of these observations. None of these healthcare workers had antibody to SARS CoV, indicating that subclinical or mild infection attributable to SARS CoV in adults is rare.

The outbreak of severe acute respiratory syndrome (SARS) at the Prince of Wales Hospital, Hong Kong, began on March 10, 2003 (*1*,*2*). Within the next 10 weeks, the hospital admitted 331 patients with SARS; 160 (48.3%) were healthcare workers (HCWs). Prince of Wales is a 1,350-bed teaching hospital with 3,711 employees, of whom 12% are physicians, 36% nurses, 11% allied health workers, and the remainder, administrative and ancillary staff. During the outbreak, many HCWs had been exposed directly or indirectly to aerosols, body fluids, secretions, and excretions of SARS patients. The clinical manifestations of SARS are well documented (*2*–*5*). However, we do not yet know the spectrum of clinical disease or whether mild or asymptomatic infections attributable to the SARS-associated coronavirus (SARS-CoV) occur. Whether subclinical infections occur and whether one may seroconvert to the SARS-CoV with minimal or no symptoms are concerns for HCWs and others.

## The Study

We performed a prospective study to determine whether asymptomatic or mild infection attributable to SARS-CoV was common in HCWs in this outbreak at Prince of Wales Hospital. When it had been established that an outbreak was occurring, a SARS screening clinic was instituted to care for hospital staff with symptoms suggestive of or suspected to be SARS. Asymptomatic staff or those without compatible symptoms were also invited to participate in this study. In late March and early April 2003, a blood sample was collected from each HCW who voluntarily participated and who wished to be tested for antibody to SARS-CoV; a second blood sample was collected 4–6 weeks later. Most of the second blood samples were collected in early May 2003, approximately 8 weeks from the first peak and 4 weeks from the second peak of admission of HCWs with SARS (Figure). Each HCW completed a questionnaire to document known direct contact with SARS patients, their body fluids, secretions, or excretions; places of duty within the hospital; and symptoms of any illness during the period between first and second blood sample collection. Additional information also included the department and the position of HCWs, so that the job nature could be delineated.

**Figure:**
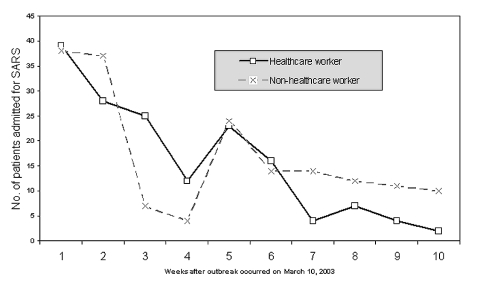
Number of patients with severe acute respiratory syndrome (SARS) admitted to Prince of Wales Hospital during the first 10 weeks of the SARS outbreak. A total of 160 healthcare workers and 171 non-healthcare workers were admitted; a second peak of admission occurred the 5th week after the outbreak started.

Immunoglobulin (Ig) G antibody to SARS-CoV was detected by an immunofluorescence assay on the basis of Vero cells infected with coronavirus isolated from a patient with SARS. We isolated this SARS-CoV and determined the complete genome sequence (GenBank accession no. AY278554). Serum samples were diluted 1:40 for antibody-screening assays. Each result was crosschecked by two experienced technicians. This immunofluorescence assay had been successfully used for serodiagnosis of SARS in patients in our hospital; titers of >320 developed in acutely ill SARS patients 4 weeks after onset of illness.

## Conclusions

Six hundred and seventy-four HCWs completed the questionnaire and had a second serum sample obtained. The mean age of these HCWs was 40 years (range 20–60), and 75% were female. HCW jobs were categorized into five groups according to those with direct patient care, namely: doctors and nurses, 28% (188); healthcare and general service assistants, 15% (104); and allied health workers, including physiotherapists, occupational therapists, and x-ray technicians, 6% (43). The remainder of staff, who did not have direct patient care, included the ancillary staff, 35% (235); pathology laboratory staff, 14% (95); and others, 1% (9 HCWs). Altogether, 43% of the HCWs reported having known direct contact with patients with SARS or their body fluids, secretions, or excretions. An additional proportion of HCWs might have had contact with patients who subsequently were confirmed to have had SARS, unknown to the HCWs. A total of 36% of the staff worked in or visited adult medical or pediatric wards with SARS patients—30% in the accident and emergency unit and 9% in the intensive-care unit—all areas at high risk for SARS within the hospital during the outbreak. Of the 674 HCWs, 353 (52%) reported mild, self-limiting illnesses during the period between the times when the first and second blood samples were collected (Table). None of the 674 HCWs was shown to have IgG antibody to SARS CoV.

**Table:** Symptoms reported by healthcare workers without SARS-CoV infection^a^

Symptom^b^	No. (%) of healthcare workers N = 353
Headache	194 (55.0)
Sore throat	174 (49.3)
Cough	140 (39.7)
Coryza	139 (39.4)
Sputum	87 (24.6)
Myalgia	83 (23.5)
Diarrhea	80 (22.7)
Dizziness	75 (21.2)
Chills/rigors	69 (19.5)
Fever	68 (19.3)

^a^SARS-CoV, severe acute respiratory syndrome–associated coronavirus. ^b^All symptoms reported were mild, self-limiting, and lasted for 1 to 2 days.

The current global outbreak of SARS is associated with a novel coronavirus, SARS-CoV, which is phylogenetically distinct from other known members of the virus family (*Coronaviridae*) and genus (*Coronavirus*) (*6*–*8*). The full clinical spectrum of this novel infection in humans has not yet been defined. Among the 674 HCWs that we examined, none showed evidence of seroconversion to SARS-CoV.

It is possible that a proportion of our study participants might not have actually been exposed to SARS-CoV. Although these participants were working in our hospital when a large number of patients with SARS were staying there, vigilant infection-control measures had been in place since the outbreak was recognized (*9*). All staff working in high-risk areas were required to wear a mask, gloves, eye goggles, and protective clothing. These measures have been shown to reduce the risk for infection (*10*).

The results of this study show that our SARS clinic successfully identified all staff with SARS-CoV infections. Alternatively, our data suggest that asymptomatic or mild forms of SARS-CoV are rare at the current point to which the virus has evolved. From the virologic viewpoint, this finding indicates that the novel coronavirus has not yet adapted to transmit among humans through asymptomatically infected hosts. This finding has important public health implications, as the level of immunity towards SARS-CoV could be very low even in members of communities that had had a large outbreak of SARS. If this is the case, a large proportion of the population remains susceptible, and another major outbreak may occur when the virus is introduced by highly infectious sources.
